# Spatial distribution and fixed-precision sequential sampling plans for *Popillia japonica* (Coleoptera: Scarabaeidae) adults in primocane raspberry: influence of foliar insecticides

**DOI:** 10.3389/finsc.2024.1465829

**Published:** 2024-10-02

**Authors:** Adam G. Toninato, Eric C. Burkness, William D. Hutchison

**Affiliations:** Department of Entomology, University of Minnesota, St. Paul, MN, United States

**Keywords:** IPM decision-making, Japanese beetle, Taylor’s Power Law, resampling, IPM, sequential sampling

## Abstract

The Japanese beetle, *Popillia japonica* Newman (Coleoptera: Scarabaeidae), an invasive species from northern Japan, was first detected in Minnesota in 1968. According to fruit growers and the Minnesota Department of Agriculture, population size and feeding damage has been an increasing concern since 2010. Based on trap-catch data, populations have recently exceeded 4,000 beetles/trap/week during July-August near raspberry fields, and can increase by an order of magnitude within 7-10 days. The primary goals of this study were to assess the spatial distribution of *P. japonica* adults in raspberry, and to develop and validate a practical fixed-precision sequential sampling plan for grower use. Taylor’s Power Law (TPL) regression was used to characterize the beetle’s spatial pattern in research plots and commercial fields, either with or without insecticide applications. We then used Green’s plan to develop an enumerative sequential sampling plan to estimate *P. japonica* density in primocane raspberry. Beetle population data were collected at two locations in southern Minnesota, including the Rosemount Research and Outreach Center, and a commercial field near Forest Lake. The TPL results, via slope comparisons, indicated no significant differences in *P. japonica* spatial pattern between insecticide treated plots versus untreated plots, or among 4 different insecticides (P>0.05). Utilizing all spatial pattern data, we characterized the distribution of *P. japonica* beetles to be highly aggregated in raspberry, with TPL slopes ranging from b = 1.38 to 1.55; all slopes were found to be >1.0. Although the slopes were not significantly different, we accounted for variability in spatial pattern by using 33 independent data sets, and the Resampling for Validation of Sampling Plans (RVSP) model to validate a sampling plan with a final average precision level of 0.25 (SEM/mean), recommended for integrated pest management (IPM) purposes. The final sampling plan required an average sample number of only 15, 1-m-row samples, while providing high relative net precision (RNP), and thus a cost-effective, efficient sample plan for growers.

## Introduction

The Japanese beetle, *Popillia japonica* Newman (Coleoptera: Scarabaeidae), is native to northern Japan and highly invasive in the U.S. The beetle was first detected in New Jersey in 1916 (1, 2), and gradually spread west and southwest. *Popillia japonica* has now been documented in at least 31 states, and was first detected in Minnesota in 1968 (3). Since then, *P. japonica* populations remained relatively small and were primarily limited to urban areas within the 7-county metro area of Minneapolis & St. Paul (4). However, since 2010, *P. japonica* outbreaks have occurred more frequently in Minnesota, and have increasingly been a concern to growers of high-value fruit crops (5, 6). *Popillia japonica* adults are known to colonize over 300 wild and cultivated plant species across 79 families, resulting in a characteristic leaf “skeletonizing” feeding pattern (2). Adult feeding during July and August in the Midwest U.S. can quickly result in high defoliation rates on economically valuable hosts, particularly linden trees, roses, and numerous field and horticultural crops (2, 3, 6, 7). In addition to direct feeding damage, invasive arthropod species can often alter the feeding patterns of native pests (e.g., 8), and consequently disrupt existing Integrated Pest Management (IPM) programs (9, 10). Specifically, *P. japonica* has been shown to exacerbate feeding injury by a native insect, the Green June Beetle (*Cotinis nitida*), that is preferentially attracted to grapes damaged by *P. japonica.* In turn, yeast production and premature fermentation impacts fruit quality (8).

Among the most attractive fruit crops in the Midwest region, raspberries, apples and wine grapes are readily damaged (2, 5, 7, 11–13). In Minnesota, both summer (floricane) and fall (primocane) raspberries are significant sources of income to growers. DiGiacomo et al. (14) recently found that Driscoll Inc. (Watsonville, CA) documented high consumer demand for raspberries in Minnesota, where the Minneapolis-St. Paul market consumed 132% more fresh raspberries than the average U.S. household. High in vitamin C and K, with high antioxidant capacity, modern raspberry cultivars have been categorized as a “super food” (15). It was recently estimated that raspberry production in Minnesota yields 3.51 million pounds of marketable product ($8.42 lb/ac), valued annually at $70.6 million (14).

Because of the value of raspberries to the Minnesota fruit industry, many fruit growers utilize a “pick your own” production system, as the need for frequent harvests is labor intensive (14). However, scheduling both pest management activities, insecticide applications, as well as safe harvest dates can be challenging (6, 14). Raspberry is a perennial crop with one of two different harvest cycles; floricane is harvested throughout the summer (primarily July-August), and primocane harvested during autumn (August-October). Primocane raspberries are most commonly grown in Minnesota and are therefore at greater risk from insect pest feeding on foliage from the mid-late portion of the growing season (5, 7, 14). *Popillia japonica* prefer feeding on raspberry leaf tissue, even in the presence of other known host plants (16). Burkness et al. (11), in a 3-year study, found that the beetle attacked raspberry fruit ~20% of time. In addition to direct damage to fruit, feeding injury to foliage may also interfere with photosynthesis and late-season fruit production (17). Primocane raspberries also endure the most feeding pressure from *P. japonica* during the transition from vegetative growth to fruiting, as sucrose and nutrients are stored for berry production during July and August (17); this period also coincides with peak flights of *P. japonica* (7, 18).

Considerable Integrated Pest Management (IPM) research to date has focused on the *P. japonica* larval (white grub) stage of the insect’s life cycle (1, 3, 19). However, relatively little applied research in the Midwest U.S. has been directed toward adult *P. japonica* ecology or an understanding of the impact on fruit crops, including yield or quality of raspberries. With concerns about the beetle’s rapid colonization of fruit crops (e.g., 5, 6, 11), and that the highly visible impact of feeding damage may increase insecticide use, growers are currently in need of an effective monitoring tool to estimate the number of *P. japonica* adults present, and thus make objective IPM decisions. The primary control practice currently includes the use of foliar insecticides soon after finding a few *P. japonica* beetles, or high levels of defoliation. In addition, the process of sampling for beetles, or estimating defoliation is a challenge, depending on the experience of growers or consultants (e.g., 1). As part of our research to develop new IPM programs, monitoring systems are needed to track beetle population trends, such as the use of semiochemical-baited traps to understand regional pest pressure (5). In addition, for individual crops such as raspberries, statistically sound sampling methods are necessary for future use with economic or “action” thresholds, to better assess if and when insecticide applications are necessary (20).

Although time-saving, sequential sampling plans are available to assist with sampling plan design (21, 22), the challenge is that the “fixed precision level” proposed for enumerative plans (e.g., 23) is actually variable for any given sampling session (or bout) (24). The desired precision level is an expectation that assumes multiple sampling sessions. To overcome this concern and to assist with sampling plan validation, Naranjo and Hutchison (25) developed the Resampling for Validation of Sample Plans (RVSP) program to allow researchers to quantify how the variability in actual precision levels obtained based on actual sampling data sets. This approach has proven useful for a variety of arthropod species, and for the validation of both enumerative (25–27) and presence-absence (28–30) sampling plans.

Given the high value of raspberries in Minnesota (14), and the subsequent use of multiple insecticides for *P. japonica*, we were motivated to develop and validate a statistically sound sampling plan for grower use. To do so, we utilized the well-known Taylor’s Power Law regression (31) to characterize the spatial pattern of *P. japonica* adults, and the RVSP resampling approach to validate a cost-effective sequential sampling plan (23, 25). To provide additional efficiency, we examined the beetle’s spatial pattern within the raspberry canopy, to minimize the area sampled.

## Materials and methods

### Sample data

The primary data sets used for sampling *P. japonica* adults, without insecticide use, were collected during the summers of 2018, 2020, and 2021, using a primocane (fall bearing) variety, ‘Heritage.’ The primary research field site was located at the Rosemount Research & Outreach Center, University of Minnesota (ROC-North, 44.71520N, -93.09744W). Raspberries were maintained using standard production guidelines for fertility, and supplemental irrigation as needed (11, 32). This site (~ 0.1 ha) consisted of several 2-row research plots (4.6m long), with rows separated by 3.1 m of turf maintained between rows to minimize erosion. In 2020, 22 data sets were collected from 5 July to 3 September; in 2021, 34 data sets were collected from 5 July to 30 August. In addition, in 2018, four high-density samples were collected in commercial fields of primocane (fall bearing) raspberries, ‘Prelude’ and ‘Nova,’ at a site near Forest Lake, MN (45.22832N, -92.89175W). These samples were collected prior to insecticide use. The field size, including both varieties was ~0.2 ha, with row spacing at 3.1m, also separated by turf between rows. In total, 60 data sets from untreated research plots and the commercial field were available for analysis. For all samples, the same 1-m row, canopy sample unit was used. On each sample date, 16 to 60 randomly selected 1-m row samples were taken using a visual whole canopy inspection, to record adult *P. japonica* densities (e.g., 5). Each data set was then used to calculate the mean and variance for sampling plan development and validation.

In addition to the ROC-North site, a second site, ROC-South (44.69104N, -93.07326W), was also established using ‘Heritage’ raspberry, where an insecticide study was conducted to assess efficacy and spatial pattern of *P. japonica* adults; this was ~4.5km south of ROC-North. ROC-south included 8 eight quadrants running east to west, where each quadrant contained 11, 3.1m rows of ‘Heritage’ raspberries separated by 3.1m turf alleys as previously described. As with ROC-north, the site was maintained using standard production guidelines for fertility, and supplemental irrigation as needed. The plots were assigned to 5 insecticide treatments, with 4 replications each, in a randomized complete block design (RCBD). The insecticide study in 2020 was conducted with sprays applied every two weeks from July 10 to August 28. Carbaryl (Sevin^®^, Monsanto, St. Louis MO), and spinosad (Entrust^®^, Corteva, Wilmington DE) were applied at rates of 32.0 and 6.0 oz of product/ac, respectively in a randomized complete block design. The zeta-cypermethrin (Mustang Maxx^®^, FMC, Philadelphia PA.) treatment, at a rate of 4.0 oz of product/ac, was applied weekly with the intent to maintain a “beetle free” zone. In 2021, the same trial was conducted at ROC-south, however, the spinosad treatment was replaced with acetamiprid (Assail^®^, United Phosphorous Inc., Bandra, East Mumbai), at a rate of 4.5oz of product/ac. In 2021, carbaryl and zeta-cypermethrin were applied at the same rates and intervals as in 2020. As with all other sampling data, the 1-m-row sample unit was used. Total data sets available for spatial pattern analysis ranged from 22-27, depending on the insecticide. In addition, a total of 16 data sets (4 data sets for each of 4 insecticides) were set aside prior to analysis, for sampling plan validation (25). Adult beetles from each study were identified by external anatomy and coloration unique to *P. japonica* (3, 19) and validated by comparisons to specimens previously deposited to the Insect Museum in the Department of Entomology, University of Minnesota.

### Spatial pattern

We examined the spatial distribution of *P. japonica* adults using Taylor’s Power Law (TPL) which is based on a logarithmic relationship between the sample variance (s^2^) and the sample mean (m) (31), such that:


$$s^{2}=a{\left(m\right)}^{b}$$

In practice, a and b are estimated by linear regression of log(s^2^) as a function of log(m), where b is the slope, and index of aggregation. In theory, the value of b is independent of mean density and is relatively “constant” for a species in a given environment (31). Among many aggregation indices, TPL has been found to provide robust estimates of spatial pattern (e.g., 22, 26, 28). During 2020-2021, a total of 60 data sets collected from raspberry plots not treated with insecticides were used to calculate the mean/variance estimates. The TPL regression was used to quantify the relationship between log variance and log mean, for 43 of the 60 population density samples. The remaining 17 samples were selected to reflect a density range from low-high, and set aside as independent data sets to be used for RVSP validation analysis (see below). The slope of the TPL regression line (*b)* is indicative of the spatial pattern of the population sampled; i.e., if *b* < 1, *b =* 1, or *b* > 1, the population can be characterized as having a uniform, random, or aggregated spatial distribution, respectively (22). In addition to the primary TPL regression (N=43), the spatial pattern for *P. japonica adults* was determined by comparing the slopes for the TPL regressions for the 4 insecticides mentioned previously, as well as the untreated plots. The total number of sample dates per insecticide ranged from 22-26, for each of the two years. All regressions were conducted using RStudio (33).

### Enumerative sequential sampling

Development of the sampling plan was based on Green’s (23) “stop-line” model to estimate population density, after a successive number of samples are taken, using the formula:


$$T_{n}\geq {\left(an^{1-b}/SE/m^{2}\right)}^{1/(2-b)}$$

where *T_n_* is the cumulative number of individuals sampled for *n* samples, *a* is the antilog of *a* from the TPL regression, *b* is the slope from TPL, SE/m = D for precision level (e.g., D=0.10, 0.25). With this plan, one continues to take successive samples until the appropriate T*_n_* is exceeded to estimate insect density, for the desired precision level.

Validation of Green’s plan was conducted by selecting 17 of the 60 sample data sets taken over two years at ROC-North prior to calculating TPL values, for use as independent data sets for validation (Naranjo & Hutchison 2007). The 17 data sets for validation were selected to represent a range of population densities from low to high. In addition, because of the potential variation in spatial pattern for insecticide treated plots, 16 sample dates were also selected from the insecticide study (4 samples for each of 4 insecticides), for a total of 33 validation data sets; for all validation data sets, the observed mean density ranged from 0.75 to 28.1 beetles per 1-m row. All validation data were then used with the TPL parameter estimates (a, b values) and Green’s plan to evaluate plan performance, via the Resampling for Validation of Sample Plans (RVSP) program (25). Using the RVSP package, during each sampling session (bout), the resampling method assumes that a unique set of random samples are taken to estimate density to mimic one taking samples at random in the field. Final plan performance is assessed after the sampling process has been repeated (simulated) 500 times for each data set. The outcomes for the actual precision levels achieved for each RVSP simulation, are compared to the desired precision, and subsequently changed, as an iterative process to achieve the final goals of D=0.10 and D=0.25, for research and IPM purposes, respectively (34). In addition, RVSP provides the predicted average population density, for comparison with actual population density, and average sample number (ASN) for each of the 500 simulation runs. Once the ASN was determined for each precision level, we also calculated Relative Net Precision (RNP), to compare the efficiency of each sampling plan based on the sampling cost and precision (27, 35),


Relative net precision = (1/(RV*c)) * 100,

where RV is relative variation (SE/mean) * 100 (34), and c is the total cost (in time) for collecting the selected sample, usually measured in person-hours.

### Canopy strata

To further improve the efficiency of sampling, and reduce sampling cost via a more refined sample unit, we conducted a study in 2021 to determine how *P. japonica* beetles were distributed within the raspberry canopy. On 10 separate sample dates in 2021, untreated ‘Heritage’ raspberry plots at ROC-North were used to visually sample the top-third (0-15 cm), middle-third (16-30 cm), and bottom-third (31-45 cm) strata of the canopy, to assess the potential for differential beetle density among strata. Nondestructive random sampling, using the 1-m-row sample unit, was conducted on each sampling date (July-August), by selecting five plants at random, within the middle 2 rows/plot, in each of four quadrats. The insect strata data was transformed using a square-root transformation [√(x+0.5)], where x is the number of adults recorded per sample per date. The mean number of beetles per 1-m-row, and SEMs, and the proportion of beetles in each strata were calculated for analysis. Prior to analysis, the proportion data were arc-sine transformed. The strata density data were analyzed using ANOVA with RStudio (33), and Tukey’s HSD test for means comparison (P=0.05).

## Results

### Spatial pattern

**Figure 1:**
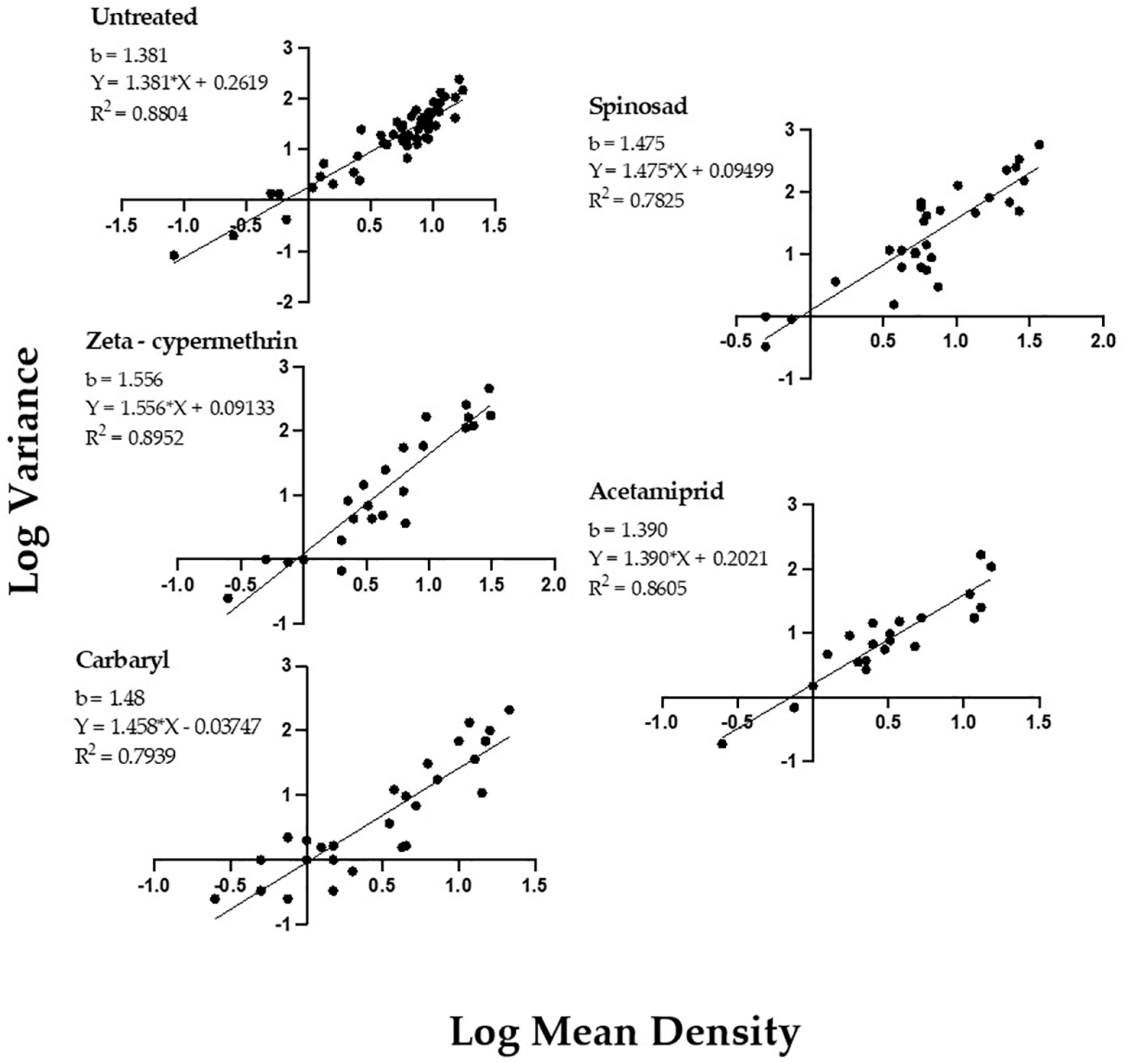
Taylor’s Power Law for *P. japonica* adult density and variance (log transformed), collected on primocane (‘Heritage’) raspberry, Rosemount MN, Forest Lake MN 2020, and Rosemount MN 2021 (see also **Table 1**).

Taylor’s Power Law regression analyses of log-variance as a function of the log-mean, for *P. japonica* adults indicated a strong positive relationship for beetle populations, as measured by the 1-m-row sample unit (**Figure 1**). The pattern was consistent regardless of the insecticide used or if the data were collected from untreated plots. For all treatment regressions, R^2^ estimates ranged from 0.79-0.89. Importantly, the TPL slope (b) values for all data sets, either from untreated or insecticide treated plots, were statistically greater than 1, (P<0.05, **Table 1**), indicating an aggregated spatial pattern for *P. japonica* adults in raspberry. We also did not find significant differences in slopes between the insecticide treated and untreated plots, nor differences among the four insecticides (P>0.05, **Table 1**).

**Table 1:** Taylor’s Power Law slope comparisons and mean density for *P. japonica* adults in raspberry, where sampling was conducted in untreated plots, and those treated with foliar insecticides, Rosemount, MN, 2021-2022.

Active Ingredient	a	*b* (± SE)^1,2^	P	R^2^	N	mean^3^ density/1-m
Zeta-cypermethrin	0.091	1.556 (± 1.49)	<0.01	0.89	24	3.45
Carbaryl	-0.037	1.450 (± 0.42)	<0.01	0.79	27	4.00
Spinosad	0.095	1.475 (± 1.13)	<0.01	0.78	27	12.88
Acetamiprid	0.202	1.390 (± 0.65)	<0.02	0.86	20	5.44
Untreated check	0.262	1.381 (± 0.28)	<0.02	0.88	43	9.55

^1^Multiple comparison test for slopes (b), with a Bonferroni correction, indicated no significant differences among slopes (P>0.75).

^2^All slopes were found to be significantly >1.0 (P<0.05), indicating an aggregated spatial distribution.

^3^Mean densities for beetles per 1-m row for both years, with the exception of spinosad data collected in 2021, and acetamiprid collected in 2022.

### Enumerative sequential sampling and validation

**Figure 2:**
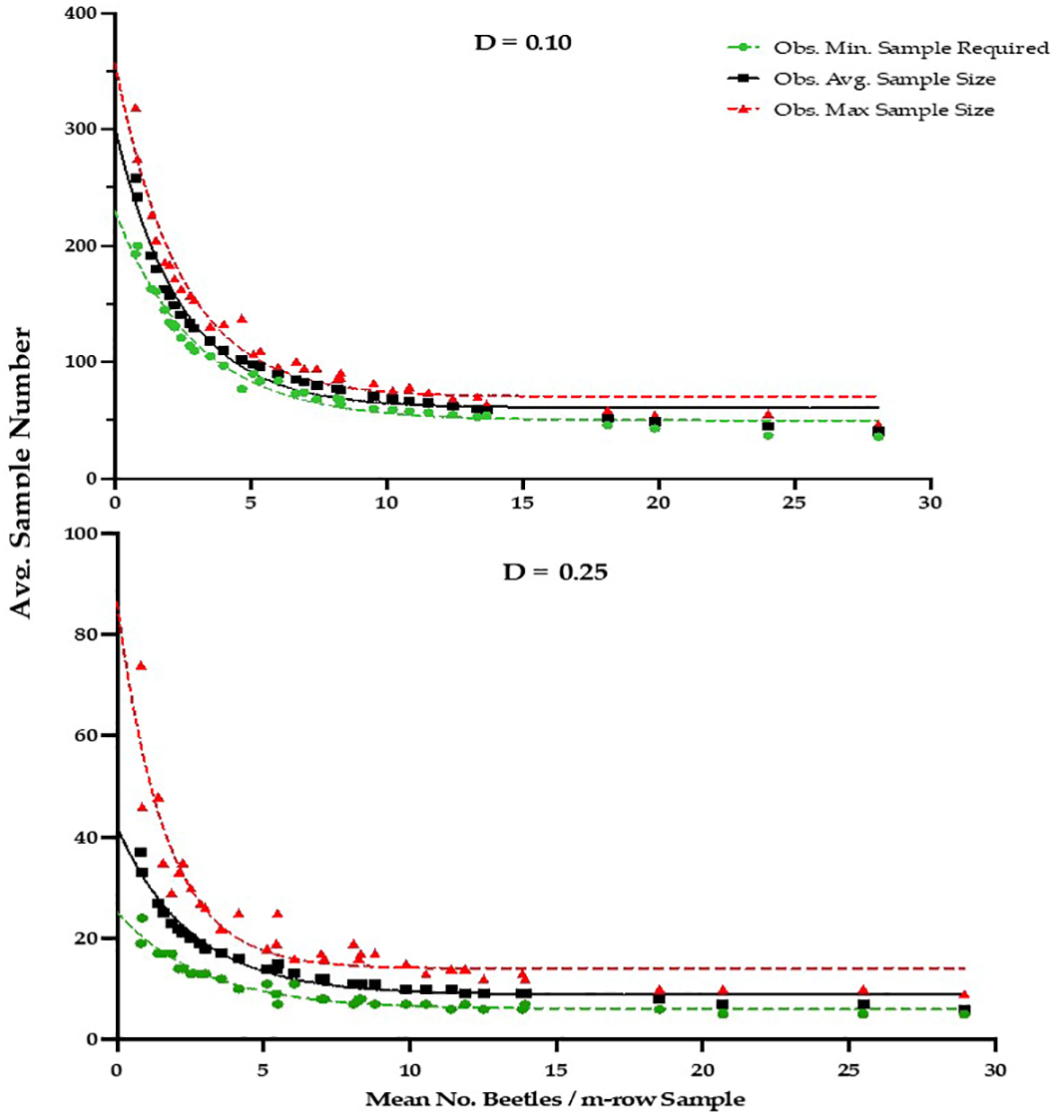
Observed Average, Minimum and Maximum Sample size results from RSVP Validation analysis for desired precision levels of D=0.10 and D=0.25, based on Green’s Sequential Sampling Plan for *P. japonica* adults, using a 1-m-row sampling unit in raspberry, Rosemount MN, 2020-2021 (see **Supplementary Table S1** for fitted equations for each precision level).

The development and validation of Green’s sequential sampling plan, via RVSP, suggested that to achieve an observed average precision level (D) of ~0.10, a high average sample size of 106 sample units are required (**Table 2**). By contrast, to achieve an average precision level (D) of ~0.25 for IPM decision-making, an average sample size of only 15, 1-m-row samples are required (**Table 3**). The initial ‘desired precision’ levels specified in the RVSP validation were higher than expected, and were therefore decreased to 0.21 and 0.08, to achieve the desired actual precision levels of 0.25 and 0.10, respectively. This option with RVSP is often necessary to adjust (fine-tune) the precision levels to determine optimum final sample size (26, 27). Based on RVSP analysis, average maximum and minimum sample sizes were 23 and 15 for a precision levels of D = 0.25. By contrast, average maximum and minimum sample sizes were 123 and 106 were necessary for a precision level of D = 0.10. The full range of expected, average sample size requirements are illustrated in **Figure 2** (see also **Supplementary Table S1**). Finally, the results for sampling efficiency, based on RNP, which includes the time to take samples for a given ASN at each precision level are shown in **Table 4**. As expected, the RNP was much higher, and most cost-effective (21.40) for the IPM-based precision level of D = 0.25.

**Table 2:** Resampling simulations used to validate a fixed precision, sequential sampling plan (23), for *P. japonica* adult density (1-m-row), by using a pre-set precision level of 0.08 (desired 0.10), via Taylor’s Power Law (*a* = 1.83, and *b* = 1.38).

Validation Data Set	Observed Mean Density	Avg. statistics for 500 sequential sampling simulations^1^						
		Mean Density	Precision			Avg. sample no.		
			Mean	Min.	Max	Mean	Min.	Max
1	0.750	0.751	0.167	0.131	0.194	258	193	319
2	0.833	0.836	0.076	0.067	0.087	242	200	275
3	1.333	1.338	0.124	0.108	0.142	192	163	226
4	1.500	1.507	0.092	0.074	0.105	180	161	205
5	1.833	1.832	0.072	0.064	0.079	163	145	186
6	2.000	1.991	0.109	0.093	0.125	157	134	184
7	2.187	2.203	0.099	0.085	0.116	149	130	172
8	2.437	2.445	0.096	0.081	0.111	141	121	163
9	2.750	2.778	0.105	0.089	0.122	133	114	157
10	2.917	2.921	0.102	0.085	0.122	129	110	154
11	3.500	3.501	0.073	0.061	0.083	118	105	131
12	4.000	4.021	0.104	0.083	0.127	110	97	133
13	4.667	4.805	0.186	0.151	0.201	102	77	138
14	5.083	5.084	0.054	0.043	0.066	98	90	107
15	5.333	5.316	0.087	0.076	0.098	96	84	110
16	6.000	6.021	0.048	0.038	0.059	90	84	96
17	6.667	6.714	0.106	0.062	0.127	85	73	101
18	6.966	6.969	0.080	0.062	0.099	83	74	95
19	7.420	7.556	0.120	0.085	0.155	80	68	95
20	8.170	8.239	0.089	0.072	0.109	77	69	86
21	8.291	8.284	0.074	0.058	0.094	76	68	87
22	8.300	8.401	0.110	0.085	0.146	76	64	91
23	9.515	9.612	0.101	0.083	0.123	71	60	82
24	10.213	10.233	0.086	0.057	0.119	69	59	76
25	10.828	10.933	0.112	0.079	0.146	67	58	79
26	11.529	11.554	0.099	0.078	0.122	65	57	74
27	12.430	12.421	0.063	0.047	0.084	62	55	69
28	13.333	13.417	0.095	0.072	0.112	60	53	70
29	13.667	13.761	0.070	0.054	0.086	59	54	64
30	18.125	18.071	0.076	0.059	0.089	52	46	59
31	19.861	19.923	0.088	0.065	0.109	49	43	55
32	24.028	24.506	0.133	0.087	0.171	45	37	56
33	28.083	28.305	0.096	0.072	0.123	41	36	47
Overall	8.01	8.07	0.097	0.076	0.117	106	91	123

1 Data sets resampled with replacement because of low mean *P. japonica* densities for some field sites (data sets 1-5); resampling conducted using Resampling for Validation of Sample Plans (RVSP) simulation software (25).

**Table 3:** Resampling simulations used to validate a fixed precision, sequential sampling plan (23), for *P. japonica* adult density (1-m-row), by using a pre-set precision level of 0.22 (desired 0.25), via Taylor’s Power Law (*a* = 1.83, and *b* = 1.38).

Validation Data Set	Observed Mean Density	Avg. statistics for 500 sequential sampling simulations^1^						
		Mean Density	Precision			Avg. Sample no.		
			Mean	Min.	Max	Mean	Min.	Max
1	0.750	0.813	0.421	0.211	0.657	37	19	74
2	0.833	0.857	0.204	0.141	0.274	33	24	46
3	1.333	1.405	0.336	0.222	0.480	27	17	48
4	1.500	1.565	0.239	0.101	0.343	25	17	35
5	1.833	1.867	0.187	0.107	0.253	23	17	29
6	2.000	2.105	0.291	0.159	0.403	22	14	33
7	2.187	2.251	0.270	0.158	0.392	21	14	35
8	2.437	2.514	0.252	0.155	0.357	20	13	30
9	2.750	2.847	0.276	0.156	0.391	19	13	27
10	2.917	3.010	0.272	0.141	0.398	18	13	26
11	3.500	3.556	0.197	0.086	0.280	17	12	22
12	4.000	4.154	0.272	0.122	0.405	16	10	25
13	4.667	5.485	0.438	0.075	0.600	15	7	25
14	5.083	5.119	0.146	0.065	0.237	14	11	18
15	5.333	5.437	0.229	0.100	0.345	14	9	19
16	6.000	6.058	0.129	0.042	0.205	13	11	16
17	6.667	6.976	0.260	0.049	0.407	12	8	17
18	6.960	7.079	0.208	0.097	0.348	12	8	16
19	7.420	8.078	0.296	0.140	0.519	11	7	19
20	8.170	8.248	0.239	0.105	0.410	11	8	16
21	8.290	8.330	0.196	0.091	0.312	11	8	17
22	8.300	8.803	0.281	0.156	0.532	11	7	17
23	9.515	9.864	0.264	0.125	0.460	10	7	15
24	10.212	10.543	0.201	0.062	0.424	10	7	13
25	10.828	11.407	0.279	0.083	0.570	10	6	14
26	11.529	11.885	0.258	0.109	0.407	9	7	14
27	12.430	12.522	0.158	0.058	0.281	9	6	12
28	13.333	13.833	0.240	0.051	0.365	9	6	13
29	13.667	13.929	0.182	0.060	0.295	9	7	12
30	18.125	18.527	0.197	0.057	0.320	8	6	10
31	19.861	20.687	0.231	0.066	0.413	7	5	10
32	24.028	25.483	0.316	0.105	0.628	7	5	10
33	28.083	28.936	0.248	0.049	0.399	6	5	9
Overall	8.010	8.310	0.249	0.106	0.398	15	11	23

1 Data sets resampled with replacement because of low mean *P. japonica* densities for selected field sites; resampling conducted using Resampling for Validation of Sample Plans (RVSP) simulation software (25).

**Table 4:** Efficiency of two fixed-precision sequential sampling plans for *P. japonica* adults in raspberry, as measured by relative net precision (RNP).

Sampling plan Precision level	ASN^1^	Avg. Sample Time (hr)^2^	Total Sample Time (hr)^3^	RNP^4^
D = 0.10	106	0.0125	1.325	7.55
D = 0.25	15	0.0125	0.187	21.40

^1^Average sample number (ASN) was estimated based on 500 iterative sampling runs (bouts), as part of the resampling analysis using RVSP (25), as per **Tables 2**, **3**. The ASN shown here is the average observed for 500 validation sampling runs.

^2^Average time to record a single 1-m-row sample (45 sec) per person, which includes the time to walk between samples (<5 sec); total sampling time = 45 sec, or 0.0125 person hr.

^3^ Time in person-hours to sample the ASN, including the time to walk between samples, for a given precision level.

^4^ Relative net precision = (1/(RV*c))*100, where RV is relative variation (D*100), and c is the total cost (time) related to collecting total samples for ASN, usually measured in person-hours.

### Canopy strata

The canopy strata distribution study, where the canopy was equally partitioned vertically across three equally spaced strata (15 cm intervals), indicated significant differences in *P. japonica* beetle density (P<0.05, **Table 5**). Over the course of 10 sample dates and using the 1-m-row sample unit, a significantly higher number of beetles were observed in the top 1/3 of the canopy (P<0.05); there were also significant differences between the mid- and bottom 1/3 strata. Likewise, the mean proportion of beetles found varied significantly by strata (P<0.05, **Table 5**), with 78.8% of the *P. japonica* adults found in the top 1/3 of the canopy. These results suggest that additional savings in sampling time could be reduced by focusing the sampling effort on the top 1/3 of the canopy.

**Table 5:** Mean (+/-SEM)^1^ number of *P. japonica* adults per m-row for three strata (15-cm intervals) within a raspberry (‘Heritage’) canopy (no insecticide sprays), Rosemount MN, 2021.

Strata	Mean No. Adults	Mean Proportion of Total
Top	9.45 (± 1.08) a	0.788 (± 2.52) a
Middle	2.10 (± 0.43) b	0.175 (± 2.18) b
Bottom	0.45 (± 0.14) c	0.037 (± 0.98) b

^1^Means followed by the same letter are not significantly different, Tukey’s HSD (P=0.05). Analysis completed based on proportion of *P. japonica* adults per stratum, using the arcsine transformation; back-transformed means presented in Table.

## Discussion

In this study we found that *P. japonica* adults were highly aggregated on primocane (fall-bearing) raspberries regardless of whether insecticides had been applied. Although the insecticides included in this study can reduce beetle densities differentially (36), our results indicate that the sampling plan presented is robust and applicable to commercial field situations for IPM decision-making. Aggregation behavior of *P. japonica* adults can be attributed to several factors related to the beetle’s ecology. First, mated females usually oviposit in moist or irrigated soil supporting turf grass or nearby pastures (1, 19), where subsequent grub (larval) populations are also known to exhibit an aggregated spatial pattern (37, 38). Secondly, *P. japonica* adults emerging from these source populations each summer are often aggregated as well. Sara et al. (39) observed an aggregated distribution of *P. japonica* adults in soybean, where beetle density was most pronounced along field edges. More recently, a similar trend was observed for *P. japonica* adults in commercial vineyards (13). Thirdly, a primary mechanism responsible for initial beetle aggregations on several plant species is attributed to the release of volatile organic compounds (VOCs) in response to beetle feeding. For example, Loughrin et al. (40, 41) quantified the attraction of *P. japonica* adult aggregations to several VOCs emanating from crab apple and wine grapes, including floral kairomones (e.g., pbenethanol, linalool) or fruit-2like volatiles [e.g., (Z)-3-hexenyl butyrate, (Z)-3-hexenyl benzoate)]. The VOCs are released soon after *P. japonica* adults initiate feeding. Although VOCs have not been documented for raspberry, a similar phenomenon could be responsible for the aggregation phenomenon observed on raspberry (see also 11). Finally, once high numbers of adult *P. japonica* adults have colonized host plants, the release of the female sex pheromone also attracts additional males to the same feeding sites (1).

An extensive entomological literature has shown that the use of Taylor’s Power Law regression provides a reliable approach to characterizing spatial pattern across a diversity of arthropod taxa (21, 24, 25, 31). Moreover, with the development of Green’s (23) sequential sampling plan for estimating population density, the slope of the TPL regression was found to be useful for developing practical sampling plans, with designated average precision levels (21, 26). However, despite the reliability of the TPL regression, and other measures of spatial aggregation, additional research affirmed that such measures serve only as initial *estimates of spatial pattern*, reflecting a continuum from random to aggregated, rather than a fixed index (24). Like other ecological parameters spatial pattern estimates are dependent on sample size, host crops, or external variables such as insecticide use. Importantly, an additional key factor affecting the final performance of a sampling plan is the stochastic nature of the sequential sampling process itself; i.e., each time a plan is implemented (sampling bout), a different set of plants are sampled and different arthropod densities are encountered, all of which yields a slightly different estimate of pest density and the final precision level obtained (21, 24, 25).

These findings prompted the development of a bootstrap, or resampling approach to develop and validate sequential sampling plans that would incorporate both sources of variation (24). As illustrated by Naranjo and Hutchison (25), the model RVSP was developed to provide a validation process for sequential sampling plans using actual insect sampling data sets, versus a theoretical distribution such as the negative binomial. The resampling approach is a form of bootstrap sampling, where independent data sets for a given species are used to assess the actual precision of a sampling plan, allowing for more flexibility in building sampling plans based on realistic spatial patterns (22, 25, 26). In addition, as an iterative process it is used to modify the pre-set precision levels, to eventually achieve the desired precision and reasonable ASN. This is particularly useful for IPM applications. By contrast, traditional plans that are not validated can lead to unnecessarily high sample sizes, that are too time-consuming (25). The simulations can also be processed in a matter of seconds. Thus, the initial pre-set precision can and should be adjusted as needed to reach the desired observed precision levels for the sampling plan to be effective. For *P. japonica* we therefore adjusted the precision levels to 0.08 and 0.21 in the final RVSP simulations to achieve the desired actual precision of D=0.10 and 0.25, respectively. The results of Green’s sequential sampling plan also indicate that as the density of *P. japonica* increases, fewer samples are required to determine adult density (**Tables 2**, **3**, **Figure 2**).

The strata study indicated that the majority of *P. japonica* adults are found in the top third of the canopy (**Table 5**). Feeding by *P. japonica* in the upper strata of crop canopies has also been documented in wine grapes (5) and soybean (42). Feeding in the upper strata of various crops has been attributed to their attraction to sunlight (UV) or the nutritional value of feeding on younger leaves (1, 11). This information will be helpful for growers and crop consultants as they can focus their crop inspections more efficiently within the upper canopy strata. Finally, although a formal time-of-day study was not conducted, we found that beetle activity was most noticeable between 11am to 5pm, and that beetle counts too early in the morning could lead to underestimates of the actual infestation levels.

To our knowledge, this is the first study to document the spatial pattern of *P. japonica* adults in raspberry. However, previous work with *P. japonica* has shown that the adults also exhibit aggregated distributions, when beetles were sampled in wine grapes where a strong “edge effect” was noted (13), or when trapping beetles in semiochemical-baited traps (5). It is also notable that the strong linear TPL relationship for *P. japonica* adults is similar to the TPL results found for other beetle species (e.g., 26). Regardless of the biological basis for aggregation pattern in the field, it is well known that the subsequent sampling plans for such species, often necessitates higher sample sizes and costs, compared to sampling plans for species characterized by random spatial patterns (25).

Our sequential sampling plan indicates that, on average, only 15 samples are necessary to estimate adult *P. japonica* population density in raspberry, when using the IPM based precision level (D) of 0.25 (**Table 3**). With a sample time of 30-45 sec, and the time to walk between samples averaging 5 sec, we assumed a final conservative estimate of 45 sec for total time to take a 1-m-row sample. Thus a sample size of 15 would equate to ~11 minutes per field site, which is a reasonable time frame for growers and crop consultants to make control decisions (**Table 4**). When beetle densities are relatively moderate to high, the sampling time will be much less (**Figure 2**). The combination of a brief sampling time and a validated precision level (0.25 for IPM), suggests a high level of efficiency in sampling adults. *Popillia japonica* often overwinter in loam-clay soils and prefer moist turf (1, 43), which is commonly grown between raspberry rows. This should inform growers that once a population is established, there will continue to be moderate to high beetle pressure in the foreseeable future making an effective sampling plan imperative. Recent studies in Minnesota suggest that *P. japonica* adult emergence begins during late June (6, 7); therefore growers and consultants in the upper Midwest region should begin sampling by mid-July to catch peak beetle activity, and begin sampling efforts.

Although the pattern for aggregation of *P. japonica* adults was consistent among all insecticides tested (b >1.0), the differences observed in population density and corresponding ASNs (**Tables 2**, **3**) was not surprising given the known differences in efficacy of insecticides for this species (36); there may also be sub-lethal or behavioral effects on the beetles in treated plots, that may also differentially affect spatial pattern. For example, Burkness et al. (36) found that zeta-cypermethrin (Mustang Maxx) consistently reduces beetle populations to <5 beetles/m-row, for up to 2 weeks following an application, resulting in several sample dates with low densities. As shown for other insect species, low densities may lead to a more random spatial pattern. Moreover, the change from aggregated to random is more of a continuum versus an abrupt change of the TPL slope from b=1.0 to b>1.0 (22).

The primary production practice for raspberries in Minnesota is the use of fall bearing varieties (14) because they yield well, and are less labor-intensive than summer raspberries that require intense labor for pruning each season. In recent years, because of the establishment of another invasive, spotted-wing Drosophila (*Drosophila suzukii*), more growers are beginning to transition toward summer bearing raspberries (14). Although our research was conducted primarily with the fall bearing ‘Heritage’ variety, we believe the proposed sampling method should be applicable to summer bearing raspberries as well. Fall and summer bearing raspberries share similar canopy growth patterns, with ample foliage produced during summer-fall growth periods, when *P. japonica* adults are most active in the Midwest region (7). However, because multiple varieties are grown in the region, further research is needed to determine the degree to which *P. japonica* adults show similar aggregation behavior, and thus changes in spatial aggregation that could affect sampling plan recommendations. In addition, more work should be done with other varieties to evaluate the degree of fruit feeding by *P. japonica* beetles, as this may require more targeted sampling on fruit and flowers versus foliage late in the season. As *P. japonica* continues to colonize crops in Europe (44–48), and global climate change continues to facilitate invasive pest expansion (49, 50), it will be critical for researchers to develop innovative monitoring tools for both surveillance and IPM applications to minimize excess insecticide use and respond to grower challenges.

In summary, our study shows that *P. japonica* adults exhibit an aggregated distribution on fall bearing ‘Heritage’ raspberries in Minnesota. Despite a high level of aggregation, the validation analysis, when using Green’s sequential sampling plan, requires an average of only 15 1-m-row samples to estimate the population density, at an average precision level (D) of 0.25, recommended for IPM decision-making (25). As with other sequential sampling plans, more samples are necessary at low densities (e.g., < 5 beetles/sample). For the high density range of 10 to 25 beetles/sample unit, the ASN continued to decline, especially for D=0.25, but ranged from 8-15 samples, respectively (**Table 3**, **Figure 2**). The sequential sampling plan for *P. japonica* on raspberry should be useful to growers and crop consultants, by providing statistically sound estimates of population density, with a reasonable sample size and cost. Finally, research is underway in Minnesota to determine economic injury levels, and a practical economic threshold for *P. japonica* in raspberry. The sampling plan can then be used in tandem with an effective economic threshold for *P. japonica* adults, to further build an IPM program for fall bearing raspberry in the Midwest U.S.

## Data Availability

The original contributions presented in the study are included in the article/**Supplementary Material**. Further inquiries can be directed to the corresponding author.
