# Supplementary material for: Spatial distribution and fixed-precision sequential sampling plans for Popillia japonica (Coleoptera: Scarabaeidae) adults in primocane raspberry: influence of foliar insecticides
Source: Front Insect Sci. 2024 Oct 2;4:1465829. doi: 10.3389/finsc.2024.1465829 (PMC11479985; doi:10.3389/finsc.2024.1465829)
Supplement: Supplementary file 1 [file Table1.docx]

**Supplemental section for Toninato et al., FIS**

This section (Table S1) includes additional information regarding non-linear regression models to characterize the Average Sample Number (ASN) relationship listed in Figure 2 of the paper. Table S1 provides the fitted non-linear functions for each of the sample size regressions.

**Table S1. Non-linear equations for the predicted sample lines, based on the simulation validation process (see Tables 2-3, Fig. 2, of main paper)***

| Avg. Sample No. | Precision | Equation | R^2^ |
| --- | --- | --- | --- |
| Obs. Min. Sample Required | 0.10 | Y = (179.67)^(-0.3363x)^+79.93 | 0.98 |
| Obs. Avg. Sample Required | 0.10 | Y = (240.3)^(-0.4119x)^+60.9 | 0.97 |
| Obs. Max Sample Required | 0.10 | Y = (287.05)^(-0.4181x)^+70.25 | 0.96 |
| Obs. Min. Sample Required | 0.25 | Y = (19.076)^(-0.3563x)^+6.064 | 0.93 |
| Obs. Avg. Sample Required | 0.25 | Y = (33.006)^(-0.4093x)^+8.894 | 0.97 |
| Obs. Max Sample Required | 0.25 | Y = (74.8)^(-0.6211x)^+13.99 | 0.89 |

***Each of the regression functions were fitted using GraphPad Prism 9.**
